# Spin-Lattice Relaxation Rates of Lipid Spin Labels as a Measure of Their Rotational Diffusion Rates in Lipid Bilayer Membranes

**DOI:** 10.3390/membranes12100962

**Published:** 2022-09-30

**Authors:** Witold K. Subczynski, Justyna Widomska

**Affiliations:** 1 Department of Biophysics, Medical College of Wisconsin, 8701 Watertown Plank Road, Milwaukee, WI 53226, USA; 2 Department of Biophysics, Medical University of Lublin, Jaczewskiego 4, 20-400 Lublin, Poland

**Keywords:** membrane fluidity, spin-lattice relaxation rate, saturation recovery EPR, spin label, stretched exponential

## Abstract

The spin-lattice relaxation rate (*T*_1_^−1^) of lipid spin labels obtained from saturation recovery EPR measurements in deoxygenated membranes depends primarily on the rate of the rotational diffusion of the nitroxide moiety within the lipid bilayer. It has been shown that *T*_1_^−1^ also can be used as a qualitative convenient measure of membrane fluidity that reflects local membrane dynamics; however, the relation between *T*_1_^−1^ and rotational diffusion coefficients was not provided. In this study, using data previously presented for continuous wave and saturation recovery EPR measurements of phospholipid analog spin labels, one-palmitoyl-2-(n-doxylstearoyl)phosphatidylcholine in 1,2-dimyristoyl-sn-glycero-3-phosphorylcholine/cholesterol membranes, we show that measured *T*_1_^−1^ values are linear functions of rotational diffusion of spin labels. Thus, these linear relationships can be used to transfer *T*_1_^−1^ values into spin label rotational rates as a precise description of membrane fluidity. This linearity is independent through the wide range of conditions including lipid environment, depth in membrane, local hydrophobicity, and the anisotropy of rotational motion. Transferring the spin-lattice relaxation rates into the rotational diffusion coefficients makes the results obtained from saturation recovery EPR spin labeling easy to understand and readily comparable with other membrane fluidity data.

## 1. Introduction

Phospholipid analog spin labels one-palmitoyl-2-(n-doxylstearoyl)phosphatidylcholine (n-PC) and stearic acid spin labels n-doxylstearic acid spin label (n-SASL) are broadly used in membrane studies. The location of the free radical fragment at different positions on the acyl chain allows profiles of certain physical properties across the lipid bilayer in a simple model [1] and complex biological membranes [2] to be obtained. Routinely, profiles of the order parameter were obtained from continuous wave (CW) electron paramagnetic resonance (EPR) spectra and interpreted as profiles of the membrane fluidity. However, these profiles show only changes of the amplitude of the wobbling motion of the acyl chain with the depth in the lipid bilayer, and thus should be considered as nondynamic [3]. Fluidity profiles of the real dynamic parameter—namely, rotational diffusion coefficients—can be obtained from computer simulation of CW EPR spectra [4]. The acyl chain of the phospholipid analog spin labels (n-PC and n-SASL) in fluid-phase membranes undergoes rapid anisotropic motion about the long axis of the spin label (with a rotational diffusion coefficient *R*_||_) and a wobbling motion of this long axis within the confines of a cone imposed by the membrane environment (with a rotational diffusion coefficient *R*_⊥_). Spectral analysis at X-band provides only one rotational diffusion coefficient, namely *R*_⊥_ [5]. The motion about the long axis of the spin label is too fast and does not affect X-band EPR spectra. Here, membrane fluidity is reported by the motion of lipid acyl chains. Simulation of the CW spectrum is not an easy process. It depends on many parameters (principal values of the g and hyperfine tensors), which should be determined for the particular environment in which the spin label is located. In addition, the CW spectra of complex biological membranes may consist of many overlapping components that reflect the various lipid environments [6] and so cannot be simulated.

Because it was accepted that *T*_1_^−1^ (spin-lattice relaxation rate) depends primarily on the rate of rotational motion of the nitroxide moiety [7,8,9,10], in our earlier papers we proposed that *T*_1_^−1^ can qualitatively describe the dynamics of the membrane environment at the depth at which the nitroxide fragment rigidly attached to the acyl chain is located. Next, we developed in greater detail this *T*_1_-sensitive EPR spin labeling method for studies of profiles of membrane fluidity [5]. We showed that the spin-lattice relaxation rate (*T*_1_^−1^) of lipid analog spin labels can be used as a convenient measure of membrane fluidity that is sensitive to the average rate of nitroxide motion. We confirmed that the measurement of *T*_1_^−1^ for a series of n-PC as a function of label position provides a fluidity profile that reflects local membrane dynamics across the membrane. Such *T*_1_^−1^ detailed profiles were obtained at X-band (9.2 GHz) [5] for 1,2-dimyristoyl-sn-glycero-3-phosphorylcholine (DMPC) membranes, both containing and not containing cholesterol (Chol). Profiles obtained from saturation recovery (SR) measurements qualitatively show the same features as the quantitative profiles obtained from the simulation of the CW spectra. Later, similar profiles were obtained at W-band (94 GHz) [11] for DMPC membranes with and without Chol, confirming that profiles obtained with SR measurement show the same features as those obtained from the simulation of the CW spectra. Also, incomplete profiles of *T*_1_^−1^ for three depths in the DMPC membrane (C5, C12, and C16), obtained with 5-SASL, 12-SASL, and 16-SASL, can be drawn based on the data obtained at S1-band (2.54 GHz), S2-band (3.45 GHz), X-band (9.2 GHz), K-band (18.5 GHz), Q-band (34 GHz), and W-band (94 GHz) [10]. In complex biological membranes, these profiles were also obtained across coexisting domains [2,12,13].

To fully appreciate *T*_1_^−1^ profiles as membrane fluidity profiles, we had to transfer these qualitative profiles so that they quantitatively report the rotational motion of the lipid acyl chain. We had to determine a procedure to transfer *T*_1_^−1^ values into spin label rotational rate values. In the present paper, we show that this is possible. Using data previously published for CW and SR EPR measurements of phospholipid analog spin labels n-PCs in DMPC/Chol membranes [5,11] at X-band and W-band and in DMPC membranes at S1-band, S2-band, X-band, K-band, Q-band, and W-band [10], we showed that the measured *T*_1_^−1^ values are linear functions of the spin label rotational diffusion. Thus, this linearity is observed for the wide range of microwave frequencies from 2.54 GHz up to 94 GHz. These relationships can be used to infer rotational rates based on *T*_1_^−1^ measurements and, thus, to obtain a precise description of membrane fluidity. The spirit of this section of the paper is to present multifrequency data empirically with minimal interpretation, very much as in papers by Hyde et al. [14] and Froncisz et al. [15], which provide *T*_1_ values for several spin labels at microwave frequencies from 2.54 GHz to 94 GHz.

Previously, we showed that profiles of *T*_1_^−1^ across lipid bilayers membranes qualitatively describe profiles of membrane fluidity. The results presented here indicate that these profiles can be transferred into quantitative profiles of spin label rotational rates. Thus, principal applications of SR in studies of biological membranes can be broadened from qualitative to quantitative measurements of membrane fluidity.

## 2. Materials and Methods

Detailed descriptions of the handling samples and performing EPR measurements were presented in our earlier publications, from which we have taken the values to use as data points in the present analysis to create appropriate calibration curves [5,11]. Briefly, the membranes used in those works were multilamellar dispersions of DMPC or DMPC/Chol (1:1 molar ratio) containing 1 mol% of n-PC (n = 5, 7, 10, 12, 14, or 16) or 1 mol% of n-SASL (n = 5, 12, or 16) (see Figure 1 in [16] for structures and the approximate location of these molecules in the bilayer). EPR measurements were performed at 27 °C for deoxygenated fluid-phase DMPC membranes, well above the main phase transition of the pure DMPC membranes [17,18]. In the presence of 50 mol% Chol, the entire DMPC membrane is in the liquid-ordered phase [18,19]. Nonlinear least squares analyses of the CW EPR spectra of n-PC were performed using the fitting program of Budil et al. [4] based on the stochastic Liouville equation developed by Freed and coworkers [20]. Analyses performed for X-band and W-band spectra are described in [5,11], respectively. SR data acquisition and processing are described in [5,11] for measurements at X-band and W-band, respectively. None of the recovery curves obtained in those studies had substantial improvements in fitting when the number of exponentials was above one, and all recovery curves were analyzed as single exponentials (the criteria for the goodness of a single exponential fit are described in [21]). Decay time constants for each sample were determined with an accuracy of more than ±3%. At W-band, phospholipid spin-label EPR spectra are in the slow-motion regime, which allows both rotational diffusion coefficients (*R*_⊥_ and *R*_||_) to be obtained from spectral analysis [11]. However, because *R*_||_ cannot be evaluated at the other microwave frequencies, we limited our presentation in the main text only to *R*_⊥_. The *R*_||_ data obtained at W-band are presented in Appendix A. An estimation of the accuracy of this motional parameter obtained from the simulation of CW spectra is difficult due to the complex interplay of parameters used for the simulation (principal values of the g and hyperfine tensors) of the spin labels. However, changes in the *R*_⊥_ by about 10% in each simulation led to an obviously worse fit. Simulation is quite sensitive to the small changes in the order parameter included in the simulation program. The accuracy of the simulation increased with the increased rotational motion of spin labels. 

The values for X-band *R*_⊥_s and the corresponding *T*_1_^−1^s were obtained from Figures 5 and 6, respectively, in Mainali et al. [5]. The W-band *R*_⊥_ values and the corresponding *T*_1_^−1^ values were obtained from Figures 5 and 6, respectively, in Mainali et al. [11]. The linear regression on the data was performed in Origin(Pro) 2019 OriginLab Corporation, Northampton, MA, USA.

Statistical differences between the slopes of linear regressions in DMPC and DMPC/Chol membranes were evaluated using a two-sample *t*-test. The *t*-values were obtained using the following equation,

*t* = (slope_1_ − slope_2_)/(sterr_1_ + sterr_2_),

where slope_1_ and slope_2_ are the slopes of the linear regression lines, and sterr_1_ and sterr_2_ are the standard error associated with the fitting. *p*-values were obtained using the *t*-values and the *t*-distribution based on appropriate degrees of freedom (number of data points minus 2).

The values of the spin-lattice relaxation rates of 5-, 12-, and 16-SASL in DMPC membranes obtained at 27 °C for deoxygenated fluid-phase DMPC membranes at S1-band (2.54 GHz), S2-band (3.45 GHz), X-band (9.2 GHz), K-band (18.5 GHz), Q-band (34 GHz), and W-band (94 GHz) were taken from Figure 4a from Ref. [10]. With the reasonable assumption that the rotational diffusion of spin labels is independent of microwave frequency, for all indicated frequencies, we used *R*_⊥_s values obtained at X-band for appropriate n-PC spin labels taken from Figure 6 of Ref. [5]. Saturation recovery EPR data acquisition and processing at all indicated microwave frequencies are described in [10]. None of the recovery curves obtained in this study had substantial improvements in fitting when the number of exponentials was above one, and all recovery curves were analyzed as single exponentials. Decay time constants were determined with an accuracy of more than ±3%. The linear regression of the data was performed in Origin(Pro) 2019 OriginLab Corporation, Northampton, MA, USA.

## 3. Results

**Figure 1:**
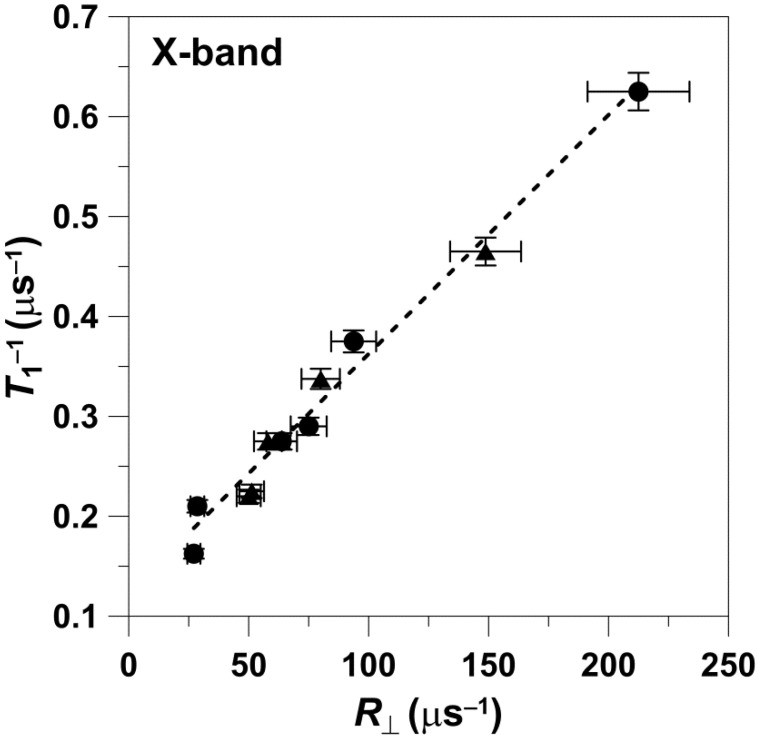
Spin-lattice relaxation rates of n-PC in DMPC (**▲**) and Chol/DMPC (**●**) membranes plotted as a function of the rate of the wobbling motion (*R*_⊥_). Measurements were performed at X-band at 27 °C for deoxygenated membranes. Values of *T*_1_^−1^ and *R*_||_ obtained for (1)—5-PC, (2)—7-PC, (3)—10-PC, (4)—12-PC, (5)—14-PC, and (6)—16-PC were taken from [5].

The data obtained at X-band are presented in Figure 1. The value of *T*_1_^−1^ measured for different n-PC spin labels increased with the increase in the rate of the rotational motion of the nitroxide moiety attached at different positions at the acyl chains. The values of *T*_1_^−1^ as a function of the values of *R*_⊥_ can be fitted with a linear equation with a slope of 0.00243 ± 0.00008 and an intercept of 0.115 ± 0.008 μs^−1^. The values that follow ± are standard errors associated with the fitting. Data points obtained for 5-, 7-, 10-, 12-, 153 14-, and 16-PC in both the pure DMPC bilayer and the DMPC bilayer containing 50 mol% Chol are linear with an R-square (adj) of 0.99.

**Figure 2:**
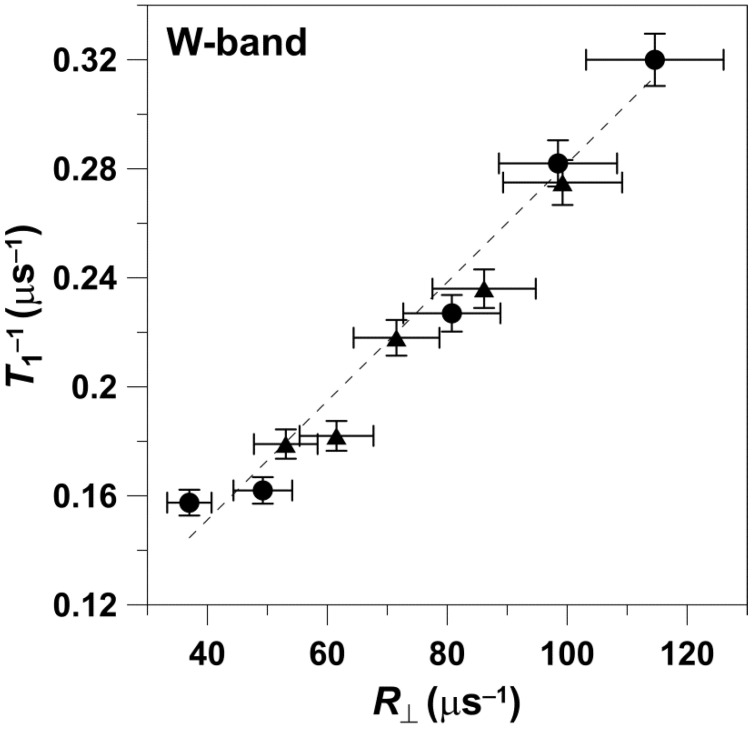
Spin-lattice relaxation rates of n-PC in DMPC (**▲**) and Chol/DMPC (**●**) membranes plotted as a function of the rate of the wobbling motion (*R*_⊥_). Measurements were performed at W-band at 27 °C for deoxygenated membranes. Values of *T*_1_^−1^ and *R*_||_ obtained for (1)—5-PC, (2)—7-PC, (3)—10-PC, (4)—12-PC, and (5)—14-PC were taken from [11]. To determine whether the local molecular environment, anisotropy of motion (order parameter) imposed by the membrane environment, or its hydrophobicity influence the linearity of *T*_1_^−1^ with respect to rotational diffusion_,_ the slopes of the linear regression lines of *T*_1_^−1^ versus *R*_⊥_ in DMPC membranes with and without Chol were evaluated for statistical difference. Table 1 lists the fitted values and the corresponding *t*- and *p*-values. All of the *p*-values were greater than the critical *p*-value of 0.05, indicating that no statistically significant difference was detected in the samples. This result leads to the conclusion that the change of *T*_1_^−1^ with respect to the rotational diffusion is not significantly affected by the local molecular environment, anisotropy of motion (order parameter) imposed by the membrane environment, or its hydrophobicity.

The data obtained at W-band are presented in Figure 2. Values of *T*_1_^−1^ versus *R*_⊥_ can be fitted with a linear equation with a slope of 0.0022 ± 0.0001 and an intercept of 0.06 ± 0.01 μs^−1^, with an R-square (adj) of 0.97. The values that follow ± are standard errors of fitting. The data points presented in Figure 2 were obtained for 5-, 7-, 10-, 12-, and 14-PC in both the pure DMPC bilayer and the DMPC bilayer containing 50 mol% Chol.

**Table 1:** Fitted slopes and corresponding standard errors of fitting were used to compute *t*- and *p*-values. *p*-values were then compared with the critical *p*-value to determine whether the slopes were significantly different.

	DMPC	DMPC/Chol	*t*-Value	*p*-Value
X-band (*T*_1_^−1^ versus *R*_⊥_)	(26 ± 2) × 10^−4^	(23 ± 1) × 10^−4^	0.96	0.39
W-band (*T*_1_^−1^ versus *R*_⊥_)	(20 ± 2) × 10^−4^	(22 ± 2) × 10^−4^	−0.38	0.73

**Figure 3:**
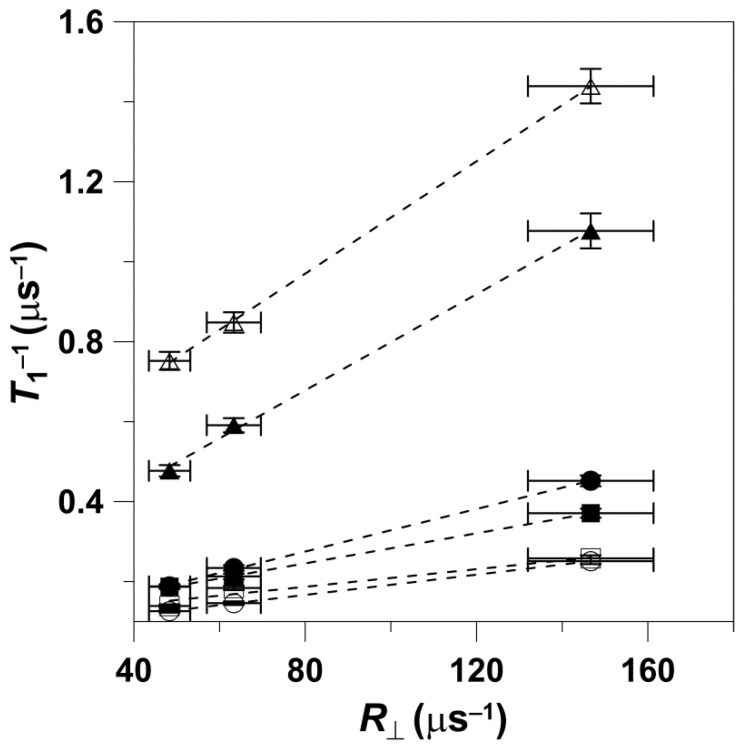
Spin-lattice relaxation rates of (1)—5-SASL, (2)—12-SASL, and (3)—16-SASL in DMPC membranes plotted as a function of the rate of the wobbling motion (*R*_⊥_). Measurements were performed at S1-band (∆), S2-band (▲), X-band (■), K-band (□), Q-band (○), and W-band (●) at 27 °C for thoroughly deoxygenated membranes. The *T*_1_^−1^ values were taken from Figure 4a of Ref. [10]. The *R*_⊥_ values obtained at X-band for the appropriate n-PC spin labels were taken from Figure 1.

**Figure 4:**
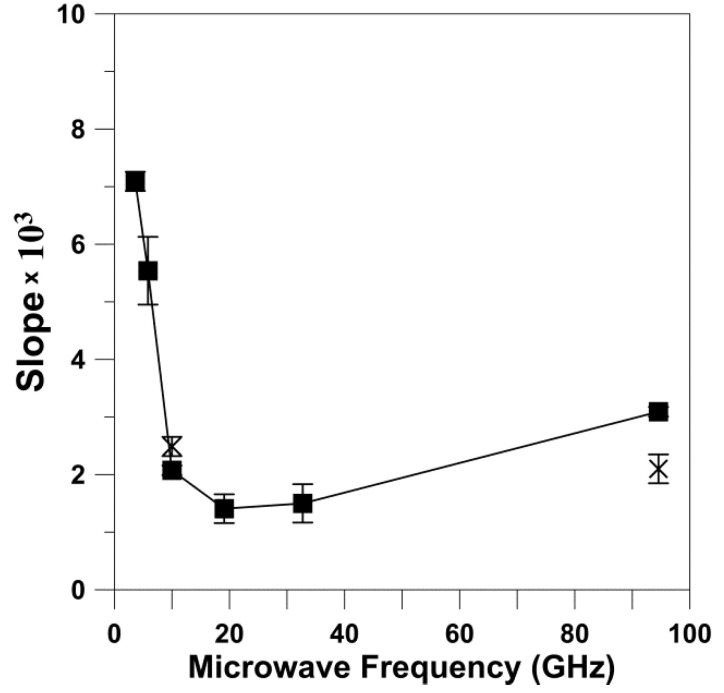
Values of slopes for the linear dependences of *T*_1_^−1^ as a function of *R*_⊥_ presented in Figure 3 (■) are plotted as a function of microwave frequency. Points for slopes obtained at X-band and W-band, taken from Figure 1 and Figure 2, are included (x). The vertical error bars indicate the magnitudes of standard error of fitting.

**Figure 5:**
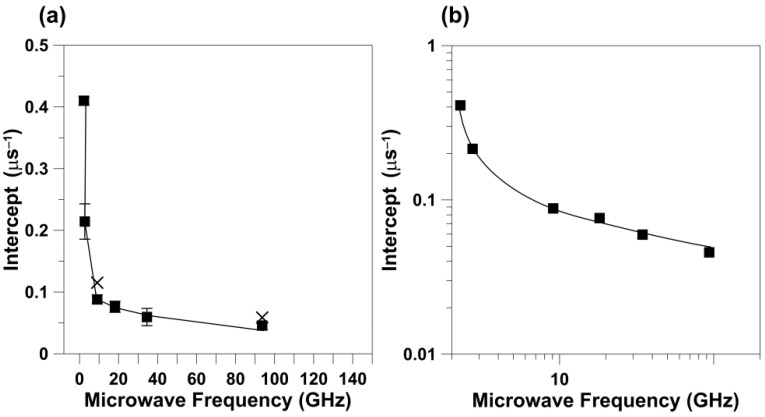
Values of intercepts for the linear dependences of *T*_1_^−1^ as a function of *R*_⊥_ presented in Figure 3 (■) are plotted as a function of microwave frequency. Points for intercepts obtained at X-band and W-band, taken from Figure 1 and Figure 2, are included (x). The vertical error bars indicate the magnitudes of standard error of fitting. (**b**) To better show that the intercept decreases monotonically with the microwave frequency, we displayed the values presented in (**a**) in the log/log scale.

We also analyzed the dependences of *T*_1_^−1^ on the rotational diffusion for frequencies other than X-band and W-band. For this analysis, we took from Figure 4a of Ref. [10] data points for the *T*_1_^−1^ of 5-, 12-, and 16-SASL in DMPC membranes obtained for deoxygenated samples at 27 °C. *T*_1_^−1^ values were obtained for all of these spin labels at S1-, S2-, X-, K-, Q-, and W-bands, thus covering the frequency region from 2.54 GHz up to 94 GHz. With the reasonable assumption that the rotational diffusion of spin labels is independent of microwave frequency, we used values obtained at X-band for appropriate n-PC spin labels (from Figure 1) as *R*_⊥_s. The cumulative results are presented in Figure 3. It is clearly shown that the dependence of *T*_1_^−1^ values as a function of the *R*_⊥_ values can be fitted with a linear equation at all microwave frequencies. The slopes and intercepts for these linear fittings are presented in Table 2. The slope significantly decreases (about seven times) between S1-band (2.54 GHz) and K-band (18.5 GHz). This tendency is broken at K-band (18.5 GHz) and Q-band (34 GHz) where the slope starts to increase, reaching a value at W-band (94 GHz) even greater that that observed at X-band. Surprisingly, the intercept decreases monotonically with the microwave frequency in all investigated frequency regions, from 2.54 GHz up to 94 GHz. The dependency of the slope and the intercept on the microwave frequency is clearly depicted in Figure 4 and Figure 5. These figures also show the slope and intercept values from Figure 1 and Figure 2. These values obtained for only X-band and W-band fit the results obtained for a broad frequency region.

## 4. Discussion

Our basic measurements (Figure 1, Figure 2 and Figure 3) were performed at physiological temperatures for phospholipid type spin labels in two significantly different anisotropic phospholipid membrane environments (pure DMPC and Chol/DMPC 1:1 membrane). The linear dependence between the relaxation rates and the rotational diffusion coefficient of these spin labels for data from both environments is clearly shown. Interestingly, a similar linear dependence between spin-lattice relaxation rates and rates of rotational motion was observed for a very different system by Yang et al. (see Figure 2c in [22]), namely for various spin-labeled sites of the T4 lysozyme protein. This result strengthens our main conclusion about the linear dependence between relaxation rates and rotational correlation rates, which can be observed for different spin labels and in different labeled systems.

Similar dependences were previously investigated but only for small spin labels in simple isotropic glass forming solvents for a broad temperature range between 100 K and 300 K [23]. Interesting data were also presented by Nakagawa [24], who investigated spin-lattice relaxation rates and rotational diffusions of small and lipid type spin labels in dispersions of hydrogenated castor oil. This triglyceride derivative forms in water dispersion bilayer membranes. Nakagawa provided changes of spin-lattice relaxation rates and rotational diffusion coefficients of indicated spin labels in hydrogenated castor oil membranes in the wide range of physiological temperatures (~20–~50 °C); however, he did not explicitly show the relation of *T*_1_^−1^s with rotational diffusion coefficients.

The data we obtained for the phospholipid bilayer membranes support the interpretation that spin-lattice relaxation rates of lipid spin labels are determined principally by the rotational motion of lipid spin labels and not by the order parameter of their restricted rotational motion in the membrane environment (see also [8]). For example, measurements with 10-PC in DMPC and Chol/DMPC membranes show practically the same *T*_1_^−1^ values of relaxation rates and practically the same values of the rotational diffusion coefficient *R*_⊥_, but the values of the order parameters are very different [5]. Thus, dynamics parameters matter.

Figure 1 includes points obtained with 10-, 12-, 14, and 16-PC. All points sit on the linear line, independent of the manner in which they were obtained for these spin labels in pure DMPC membrane or Chol/DMPC 1:1 membrane. The polarity of the local environments of the nitroxide moieties of these spin labels in the pure DMPC bilayer is close to the polarity of 1-propanol and ethanol (ε = 20–25) and in the Chol/DMPC membranes is close to that of dipropylamine (ε~3) [25]. This indicates that the polarity of the local environment does not affect the linear dependence of the *T*_1_^−1^ values on either *R*_⊥_.

The results obtained also indicate that the local molecular environment of the spin label does not affect the linearity. In pure DMPC, the acyl chains of n-PC spin labels are in contact with similar, saturated flexible acyl chains of DMPC, while in Chol/DMPC membranes, the n-PCs chains are in contact with flexible DMPC chains as well as rigid plate-like Chol structures. The contact with the rigid plate-like Chol structure reaches the depth of the ninth carbon. At deeper locations, the nitroxide moiety is again surrounded by saturated hydrocarbon chains. Even data with 16-PC, which is located at a depth exceeding the lens of the acyl chains of DMPC (14 carbons in the chain), fulfils the linearity in DMPC and Chol/DMPC membranes. The nitroxide moiety of this spin label should be in the membrane center or may even “overshoot” into the opposite leaflet of the membrane.

On the basis of the data discussed above, we can conclude that the linear correlation between the *T*_1_^−1^ and the *R*_⊥_, which was observed for phospholipid spin labels in fluid-phase lipid bilayer membranes, is independent of the structure of the environment of the spin label, the polarity of the local nitroxide environment (which changes with the depth and Chol content in the membrane), and the anisotropy of rotational motion (independent of the order parameter). All of this supports the statement that calibration curves obtained for n-PC in fluid-phase DMPC and Chol/DMPC membranes can be used for different fluid-phase phospholipid bilayer membranes. It should be noted that these calibration curves will not apply to more complex systems such as those containing phase-separated lipid domains or integral membrane proteins that affect spin label distribution and mobility.

The linear dependence between spin-lattice relaxation rates and rates of rotational motion obtained by Yang et al. [22] for various spin-labeled sites in the α-helix of T4 lysozyme protein differ from those obtained for phospholipid spin labels in lipid bilayer membranes. It follows that calibration curves differ for different spin labels and different environments. Different spin labels refer to the different nitroxide fragments (with different principal values of the g and hyperfine tensors) and different attachments of these fragments to the parent molecule. Our results show that n-PC and n-SASL form one consistent class of spin labels. Our results also indicated that lipid bilayer membranes form consistent environments for applications of calibration curves. Probably, other spin labels with the same nitroxide moiety structure and the same attachment to the parent molecule (protein) can form another consistent class. However, these are our predictions, and the subjects must be investigated in more detail.

The major conclusions from measurements at different microwave frequencies for phospholipid spin labels in lipid bilayer membranes can be summarized as follows: (1) For the wide range of microwave frequencies, from 2.54 GHz and up to 94 GHz, we observed the linear relation between *T*_1_^−1^ and the *R*_⊥_ (Figure 3). (2) The slope of this dependence decreases significantly (about seven times) between 2.54 GHz and 18.5 GHz. This tendency is broken at K-band and Q-band, and at W-band the slope increases up to the value comparable or even greater than that observed at X-band (Figure 4). (3) The intercept decreases monotonically with the microwave frequency (Figure 5) without breaking this decreasing tendency at Q-band, as observed for the change in the slope.

Interestingly, the dependence of *T*_1_^−1^ as a function of *R*_⊥_ (Figure 1 and Figure 2) shows practically the same slope when measured at X-band and at W-band. These results can be clearly understood only when looking at Figure 4 and Figure 5, where results for the broad microwave frequency region are presented. The results presented in Figure 1 and Figure 2 were obtained on both arms of the microwave frequency dependence, which show the minimum at K-band and Q-band (see the points for slopes taken from Figure 1 and Figure 2 and included in Figure 4). Similarly, *T*_1_^−1^ values measured at X-band and W-band are close to each other; even so, they show a minimum at Q-band [10]. As follows from these data, choosing only two frequencies for data analysis can lead to the wrong conclusions.

A remarkable observation is that the values of the slope (Figure 4) but not the intercept (Figure 5) obtained at 94 GHz depart strongly from the trend observed using data obtained at lower frequencies. The discovered break in the decreasing trend of the slope versus microwave frequency is like one we previously observed for relaxation rates versus microwave frequency for small spin labels in water [15] and lipid spin labels in membranes [10], which clearly showed that the minimum of this dependence is at Q-band. At microwave frequencies greater than 34.6 GHz, the spin-lattice relaxation rates increase and their values at W-band become close to those at X-band. Similarly, Hofbauer et al. [26] report unexpectedly short *T*_1_ values for 1 mM solution of TEMPO in a 10% glycerol–water mixture at W-band and suggest a specific frequency-dependent relaxation mechanism involving dynamic modulation of the g-value. All of these experimental data are in contrast with the theoretical prediction of Mailer at el. [8], which indicates that *T*_1_^−1^s measured at W-band should be about four times smaller than those measured at X-band. Theory predicts *T*_1_^−1^ changes up to 34.6 GHz (Q-band) [14] but not at greater microwave frequencies. In our explanations, we assume that the dynamics of spin labels (i.e., their rotational diffusion) are independent of microwave frequency, so they are the same for all frequencies used (from 2.54 GHz to 94 GHz). With that most reasonable assumption, it is easy to explain why the slope of the relation between *T*_1_^−1^ and *R*_⊥_ changes with frequency: it follows the changes in *T*_1_^−1^. The unexpected break in the trend of decreasing relaxation rates versus microwave frequency at Q-band requires further investigation and theoretical explanation.

Our primary intention in the present paper and those previously published [11,14,15], where we provided *T*_1_ values from 2.54 to 94 GHz, is to publish only raw new multifrequency experimental findings and refrain from theoretical explanation of the unexpected break observed at Q-band. However, we believe that the results presented will help to better explain the observed anomaly. We hope to shed some light on the mechanisms responsible for the anomaly we observed based on the behavior of the values of intercepts for the linear dependences of *T*_1_^−1^ as a function of *R*_⊥_ (Figure 5 and Table 2). The intercepts indicate the values of *T*_1_^−1^ when the rotational diffusion of spin labels is extrapolated to zero and no longer affects their spin-lattice relaxation rates. In these conditions, the intramolecular motion forms the major relaxation pathway for spin labels. As shown in Figure 5, the intercept decreases monotonically with the microwave frequency without breaking this decreasing tendency at Q-band, as observed for the change in the *T*_1_^−1^ and the slope. These results strongly suggest that the rotational motion of spin labels is involved in the mechanism responsible for the observed anomaly.

Inversion recovery (IR) is another pulse technique that can be used (in addition to SR) to measure *T*_1_. The disadvantage of the IR technique is that spectral diffusion processes with time constants that are shorter than *T*_1_ contribute to the recovery curve. Also, there are limitations on the length of the saturating microwave pulse. In the SR spectrometer, the saturating microwave pulse can be made as long as is required to saturate the spectral diffusion processes. Most significant for our research is that SR can be used even when *T*_2_ is so short (for narrow EPR lines), the condition at which IR cannot measure *T*_1_. In our experiments with lipid nitroxide spin labels in fluid phase membranes we most often deal with narrow EPR spectra. For more details on comparison of SR with IR see Section 4.4 in [27].

## Figures and Tables

**Table 2:** Values of the slopes and intercepts for the linear fittings of *T*_1_^−1^ as a function of *R*_⊥_ presented in Figure 3. The values following ± are the standard error of fitting.

	Intercept (μs^−1^)	Slope	R^2^
S1-band	0.406 ± 0.006	(71 ± 1) × 10^−4^	1.00
S2-band	0.215 ± 0.003	(58 ± 3) × 10^−4^	0.99
X-band	0.087 ± 0.002	(21 ± 1) × 10^−4^	1.00
K-band	0.078 ± 0.009	(14 ± 1) × 10^−4^	0.99
Q-band	0.059 ± 0.002	(15 ± 2) × 10^−4^	0.97
W-band	0.042 ± 0.003	(31 ± 1) × 10^−4^	1.00

**Figure A1:**
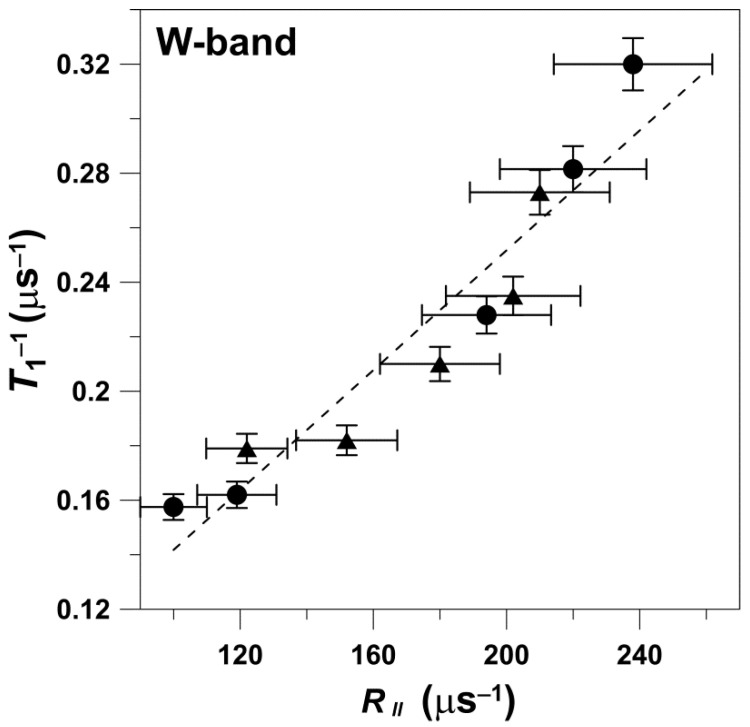
Spin-lattice relaxation rates of n-PC in DMPC (▲) and Chol/DMPC (●) membranes plotted as a function of the rate of the rapid anisotropic motion about the long axis of the spin label (*R*_||_). Measurements were performed at 27 °C for deoxygenated membranes. Values of *T*_1_^−1^ and *R*_||_ obtained for (1)—5-PC, (2)—7-PC, (3)—10-PC, (4)—12-PC, and (5)—14-PC were taken from [11].

## Data Availability

Not applicable.

## Appendix A

### Appendix A.1. Approaches to Obtain T_1_ and R_∥_ Data at W-Band

These data were obtained for DMPC or DMPC/Chol (1:1 molar ratio) multilamellar liposomes containing 1 mol% of one-palmitoyl-2-(n-doxylstearoyl)phosphatidylcholine (n-PC, n = 5, 7, 10, 12, or 14). Nonlinear least squares analyses of the CW EPR spectra of n-PC were performed using the fitting program of Budil et al. [4] based on the stochastic Liouville equation developed by Freed and coworkers [20], as described in [11]. SR data acquisition and processing are described in [11]. None of the recovery curves obtained in those works had substantial improvements in fitting when the number of exponentials was above one, and all recovery curves were analyzed as single exponentials. Decay time constants were determined with accuracy better than ±3%. Estimation of the accuracy of motional parameter (*R*_||_) obtained from the simulation of CW spectra at W-band is difficult due to the complex interplay of these parameters. However, changes in *R*_||_ by ~10% lead to an obviously worse fit. Simulation is quite sensitive to the small changes in the order parameter included in the simulation program. The accuracy of the simulation increases with the increased rotational motion of spin labels.

The W-band *R*_||_ values and the corresponding *T*_1_^−1^ values were obtained from Figures 5 and 7, respectively, in Mainali et al. [6]. The linear regression on the data was performed in Origin(Pro) 2019 OriginLab Corporation, Northampton, MA, USA.

Statistical differences between the slopes of linear regressions in DMPC and DMPC/Chol membranes were evaluated using a two-sample *t*-test. The *t*-values were obtained using the following equation,

*t* = (slope_1_ − slope_2_)/(sterr_1_ + sterr_2_),

where slope_1_ and slope_2_ are the slopes of the linear regression lines, and sterr_1_ and sterr_2_ are the standard error associated with the fitting. *p*-values were obtained using the *t*-values and the *t*-distribution based on the appropriate degrees of freedom (number of data points minus 2).

### Appendix A.2. Results and Discussion

The data obtained at W-band are presented in Figure A1, where the *T*_1_^−1^ values are plotted as a function of the *R*_||_ values and can be fitted with a linear equation with a slope of 0.0011 ± 0.0001, an intercept of 0.03 ± 0.02 μs^−1^, and R-square (adj) of 0.89. The values that follow ± are standard errors associated with the fitting. The data points presented in Figure A1 were obtained for 5-, 7-, 10-, 12-, and 14-PC in both the pure DMPC bilayer and the DMPC bilayer containing 50 mol% Chol.

The slopes of the linear regression lines of *T*_1_^−1^ versus *R*_||_ in DMPC membranes with and without Chol were evaluated for statistical difference. Table A1 lists the fitted values and the corresponding *t*- and *p*-values. The *p*-value was greater than the critical *p*-value of 0.05, indicating that no statistically significant difference was detected in the samples. This result confirms the conclusion stated in the main text that the change of *T*_1_^−1^ with respect to the rotational diffusion is not significantly affected by the local molecular environment, the anisotropy of motion (order parameter) imposed by the membrane environment, or its hydrophobicity.

**Table A1:** Fitted slopes and corresponding standard errors of fitting were used to compute *t*- and *p*-values. *p*-value were then compared with the critical *p*-value to determine if the slopes are significantly different.

	DMPC	DMPC/Chol	*t*-Value	*p*-Value
W-band (*T*_1_^−1^ versus *R*_||_)	(10 ± 3) × 10^−4^	(11 ± 2) × 10^−4^	−0.41	0.71
